# Highly Uniform Multi-Layers Reduced Graphene Oxide/Poly-2-aminobenzene-1-thiol Nanocomposite as a Promising Two Electrode Symmetric Supercapacitor under the Effect of Absence and Presence of Porous-Sphere Polypyrrole Nanomaterial

**DOI:** 10.3390/mi14071424

**Published:** 2023-07-14

**Authors:** Mohamed Rabia, Asmaa M. Elsayed, Ahmed M. Salem, Maha Abdallah Alnuwaiser

**Affiliations:** 1 Nanomaterials Science Research Laboratory, Chemistry Department, Faculty of Science, Beni-Suef University, Beni-Suef 62514, Egypt; 2 TH-PPM Group, Physics Department, Faculty of Science, Beni-Suef University, Beni-Suef 62514, Egypt; 3 Department of Chemistry, College of Science, Princess Nourah bint Abdulrahman University, P.O. Box 84428, Riyadh 11671, Saudi Arabia

**Keywords:** poly-2-aminobenzene-1-thiol, reduced graphene oxide, polypyrrole, pseudo capacitor

## Abstract

A uniform and highly porous reduced graphene oxide/poly-2-aminobenzene-1-thiol multi-layer (R-GO/P2ABT-ML) nanocomposite was synthesized and characterized. The uniform layer structure and porosity of the nanocomposite, combined with its conductivity, make it an ideal candidate for use as a pseudo supercapacitor. To enhance the capacitance behavior, a porous ball structure polypyrrole (PB-Ppy) was incorporated into the nanocomposite. When tested at 0.2 A/g, the capacitance values of the R-GO/P2ABT-ML and R-GO/P2ABT-ML/PB-Ppy were found to be 19.6 F/g and 92 F/g, respectively, indicating a significant increase in capacitance due to the addition of PB-Ppy. The energy density was also found to increase from 1.18 Wh.kg^−1^ for R-GO/P2ABT-ML to 5.43 Wh.kg^−1^ for R-GO/P2ABT-ML/PB-Ppy. The stability of the supercapacitor was found to be significantly enhanced by the addition of PB-Ppy. The retention coefficients at 100 and 500 charge cycles for R-GO/P2ABT-ML/PB-Ppy were 95.6% and 85.0%, respectively, compared to 89% and 71% for R-GO/P2ABT-ML without PB-Ppy. Given the low cost, mass production capability, and easy fabrication process of this pseudo capacitor, it holds great potential for commercial applications. Therefore, a prototype of this supercapacitor can be expected to be synthesized soon.

## 1. Introduction

Over the past few years, many serious problems have been affecting the whole of humanity and will continue into the future. One of the most important of these problems is the depletion of natural resources, so it is important to search for alternative sources of clean and renewable energy, especially due to the high demand for it because of technological progress. The development in electrochemistry led to the development in the production of electrochemical capacitors, and the focus of this study is of how energy can be stored and converted [1,2,3].

In the recent past, as a result of the need to store energy for a large number of different devices, capacitors with high speed and high power and some hybrid electrical devices were produced [4,5]. The energy storage mechanism is either by absorbing ions in the electrode (electrolyte), or by reversible high-speed faradic reactions. This type of capacitor is called a pseudo-capacitor. Excellent extensive studies have been carried out on different types of materials to find out whether these materials are suitable as electrodes for a high-speed capacitor. After a large number of experiments and research, researchers have concluded that graphite produces promising results and is the focus of extensive research. This is because it is carbon as a form of bonded carbon with special electronic and mechanical properties, as well as being two-dimensional and containing a large surface area at room temperature [6,7]. Advancements in nanoparticle technology have significantly improved the efficiency of supercapacitors by enhancing their electrochemical performance. These advancements primarily involve increasing the surface area of the electrodes, leading to enhanced charge storage and improved electrolyte diffusion within the electrodes. By utilizing nanoparticles, the available surface area for ion adsorption and charge storage is greatly increased, resulting in higher energy storage capacity and faster charge/discharge rates. These developments have contributed to the remarkable progress and efficiency improvements observed in supercapacitors. Graphite nanomaterials and carbon nanotubes are typical electrodes. Commercial electrolytes combined with carbon materials have dual advantages related to cost effectiveness and large production, which has been the motivation behind the use of these materials for commercial supercapacitor production [8,9,10].

The discovery of electrolytes with large pores and electrodes with high capacity has enabled them to function effectively within integrated systems. These developments have enabled the design and implementation of modern and sophisticated energy storage systems [2,11,12]. The electrodes consist of carbon material with so-called claws, a large surface area, high density, and a good conductor of electricity [13].

In recent years, great interest has been paid to electrodes produced from graphite and its derivatives due to its excellent chelating, mechanical, and electrical properties in addition to its electrochemical stability. The utilization of R-GO in supercapacitors is a highly promising concept due to its exceptional properties. R-GO offers a high surface area, facilitating efficient ion adsorption and subsequent charge storage. Additionally, R-GO exhibits excellent electrical conductivity, making it an ideal material for supercapacitor electrodes. The cost-effectiveness and favorable electrical properties of R-GO provide strong motivation for researchers to employ it in supercapacitor applications [14,15,16]. These catalytic characteristics have resulted in these electrodes being used in a wide range of current and potential applications and led to the improvement of graphite derivatives and the production of new compounds [17].

Polymer nanomaterials represent a novel and great choice for supercapacitors. Conductive polymers such as (polythiophene, polypyrrole, polyaniline and its derivatives) are considered promising materials for charge storage instead of other oxide or sulfide materials [18,19]. Hat et al. [20] studied the incorporation of carbon materials with polymer materials and they achieved C_S_ of 0.03 F/cm^−2^. Some recent studies have advised and promoted the incorporation of polymer as a composite for supercapacitor application; the fabricated device combines the electrical and then the charge storage behavior of all the incorporated materials [21,22,23]. 

Herein, this study presents the synthesis and characterization of a uniform and highly porous R-GO/P2ABT-ML nanocomposite, along with the synthesis and characterization of PB-Ppy. These materials were used to fabricate a supercapacitor device with two symmetrical electrodes. The device’s performance was evaluated using charge and CV studies to determine the CS and E parameters to evaluate the capacitance performance. The unique properties of this device make it a promising candidate for various industrial applications.

## 2. Experimental Section

### 2.1. Materials

Graphite powder and KMnO_4_ were acquired from Pio-Chem Co. in Giza, Egypt, while Nafion and pyrrole were obtained from Sigma Aldrich in the St. Louis, MO, USA and Darmstadt, Germany, respectively. The 2-aminobenzene-1-thiol was purchased from Merk Co in Germany. HCL, H_2_O_2_, and K_2_S_2_O_8_ were obtained from El Naser Co. in Egypt.

### 2.2. Brocken-Ball Shape P2ABT Preparation

The preparation of broken-ball shaped P2ABT involved the oxidative polymerization of 2-aminobenzene-1-thiol using K_2_S_2_O_8_. The monomer (0.12 M) was dissolved in HCl (0.6 M), which acted as both an acid medium and solvent. The addition of the oxidant triggered a rapid polymerization process, resulting in the formation of BB-P2ABT with a unique morphology characterized by broken-ball shapes.

### 2.3. R-GO Preparation

The preparation of GO involved using the modified Hummer method [24], where KMnO_4_ was used as an oxidant to oxidize graphite sheets in a highly concentrated acid medium. A total of 1.0 g of graphite was used as the carbon source in the reaction. This process led to the production of graphene oxide sheets and the formation of GO. R-GO was prepared by reducing GO (weak oxidant) using 2-aminobenzene-1-thiol (reductant). GO was mixed with this monomer for 1 h, resulting in the formation of R-GO, in which the oxidizing groups of the GO facilitated its reaction with 2-aminobenzene-1-thiol.

### 2.4. R-GO/P2ABT-ML Nanocomposite

To prepare the R-GO/P2ABT nanocomposite, a suspension of 20 mL of GO (11 mg/mL) with the monomer was created and left for 1 h. The polymerization process was completed by adding K_2_S_2_O_8_ (as mentioned earlier), to form the R-GO/P2ABT nanocomposite. The composite was then cleaned and dried, resulting in a unique morphology for the R-GO/P2ABT-ML.

### 2.5. PB-Ppy Preparation

PB-Ppy was prepared by oxidizing pyrrole monomer using K_2_S_2_O_8_ in an acid medium (0.5 M HCl). The resulting PB-Ppy exhibited a unique morphology characterized by porous ball-shapes, indicating the presence of a large surface area and active sites that make it suitable for coating with additional materials.

### 2.6. Supercapacitor Fabrication

To synthesize the R-GO/P2ABT-ML nanocomposite supercapacitor, a paste composed of 0.04 g nanocomposite and 0.005 g graphite was loaded onto each Au-plate. A binder solution containing 0.1 mL of Nafion and 0.75 mL of ethanol was used. PB-Ppy (0.01 g) was added to the nanocomposite paste to enhance its performance. A Whatman paper saturated with 1.0 M HCl was used to prevent short-circuiting between the plates. The electrochemical reactions of charge, cyclic voltammetry, EIs, and lifetime were tested using a CHI608E power station. The C_S_ and E values were calculated to determine the efficiency of the device.

### 2.7. Characterization

The materials were characterized using various analytical techniques. X-ray diffraction (XRD) was performed using X’Pert Pro (Holland) to analyze the crystal structure of the materials. Fourier transform infrared spectroscopy (FTIR) was conducted using a Jasco apparatus (Kyoto, Japan) to study the functional groups present in the samples. Energy-dispersive X-ray spectroscopy (EDX) was performed to determine the elemental composition of the samples using AXIS-NOVA, UK. The 3D morphology was also determined using SEM ZEISS, Germany. Transmission electron microscopy (TEM) 2100 (USA) was used to study the topography and 2D morphology of the samples.

## 3. Results and Discussion

### 3.1. Analyses

**Figure 1:**
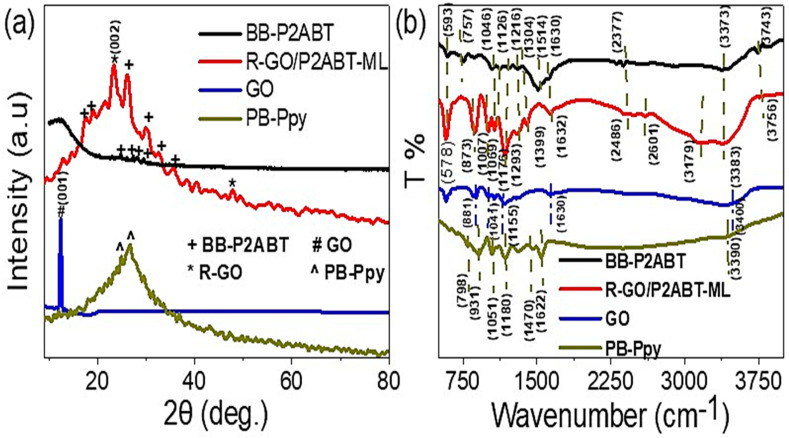
(**a**) XRD and (**b**) FTIR of PB-Ppy, BB-P2ABT, and R-GO/P2ABT-ML nanocomposite.

The XRD analysis was conducted on PB-Ppy, GO, BB-P2ABT, and R-GO/P2ABT-ML nanocomposite materials to study their crystalline behavior. The results, as shown in Figure 1, indicate that both PB-Ppy and BB-P2ABT polymers have a crystalline structure, with peaks in the 2 Theta region between 24.3 and 29.5°, suggesting their potential for energy storage applications in supercapacitors. To improve the crystallinity of BB-P2ABT, a R-GO/P2ABT-ML nanocomposite was prepared, which exhibited a higher degree of crystallinity compared to BB-P2ABT alone. The peaks related to BB-P2ABT became more pronounced, and the peaks related to GO appeared at 12.9° for the growth direction (001), along with an additional peak at 47.8° for R-GO materials. The crystal size (D) of BB-P2ABT within the composite was estimated to be 14 nm, while the crystal size of R-GO was found to be 201 nm, using Scherrer’s equation (Equation (1)) [25,26], which relates the full width half maximum (ß) and the 2 Theta angle (θ):

(1) D=0.94λ/ßcosθ

The functional groups of PB-Ppy, GO, BB-P2ABT, and R-GO/P2ABT-ML nanocomposite materials were determined using FTIR, as demonstrated in Figure 1b. Table 1 summarizes the functional groups and their corresponding band positions for each material. The PB-Ppy showed a main ring function group at 1545 cm^−1^, while GO exhibited C-O, C=O, and O-H oxidizing groups at 1155 and 1049, 1630, and 3400 cm^−1^, respectively. The characteristic functional groups of BB-P2ABT were identified at 3743, 3373, and 1304 cm^−1^ for N-H, S-H, and C-N, respectively. After the incorporation of R-GO in the nanocomposite, some shifts to the blue or red side were observed, indicating changes in the functional groups due to the presence of R-GO [27].

**Figure 2:**
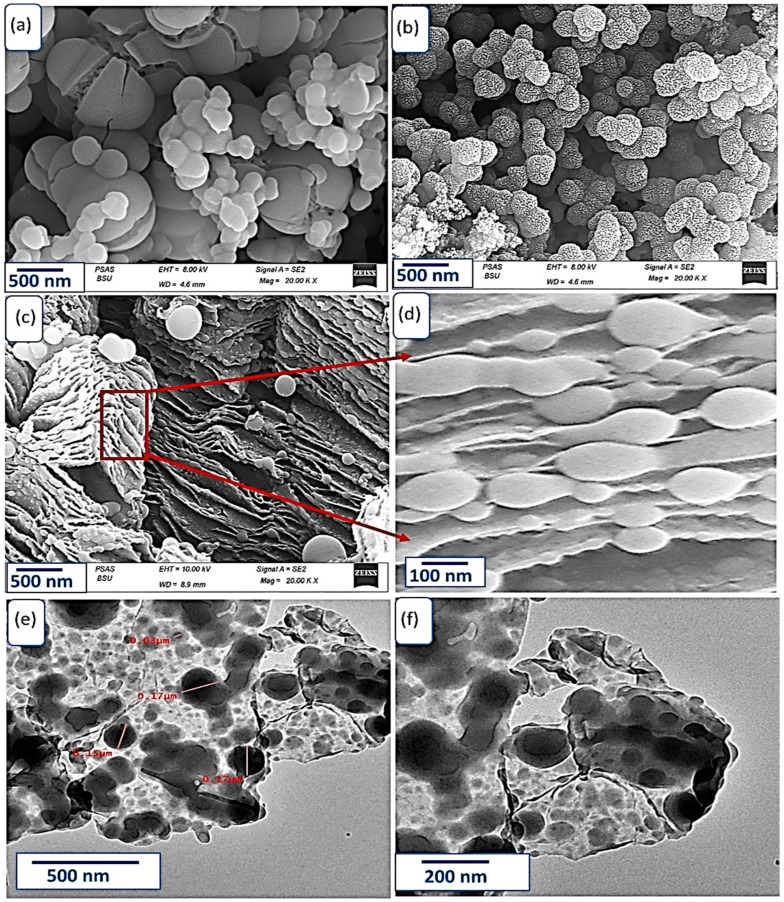
SEM of (**a**) BB-P2ABT, (**b**) PB-Ppy, and (**c**,**d**) R-GO/P2ABT-ML nanocomposite. (**e**,**f**) TEM of R-GO/P2ABT-ML nanocomposite at different magnification.

The morphology of materials can greatly affect their electrical properties, so SEM and TEM were used to analyze the topography and morphology of the materials, as shown in Figure 2. BB-P2ABT material (Figure 2a) has a broken ball-like shape with a diameter ranging from 125 to 500 nm, which provides a large surface area for the material and allows for the formation of composites with other materials. PB-Ppy (Figure 2b), on the other hand, had a uniform diameter of 150 nm and exhibited a great porosity with a wrinkle behavior on the surface. These morphological properties are important for optimizing the performance of the materials [33,34,35].

The BB-P2ABT and R-GO composite displays a new morphological behavior with highly uniform multilayers (Figure 2c,d), where the spherical bead-like shape decorates these layers uniformly. The high porosity of the composite allows for highly active sites for charge storage [36], making it suitable for supercapacitor applications. The TEM images (Figure 2e,f) confirmed the morphology of the composite, with spherical shapes (dark color) decorating R-GO sheets (grey color). Therefore, the excellent morphology of R-GO/P2ABT-ML composite indicates its potential for supercapacitor applications, due to its charge storage behavior.

### 3.2. Electrochemical Behavior of the Fabricated R-GO/P2ABT-ML Supercapacitor

**Figure 3:**
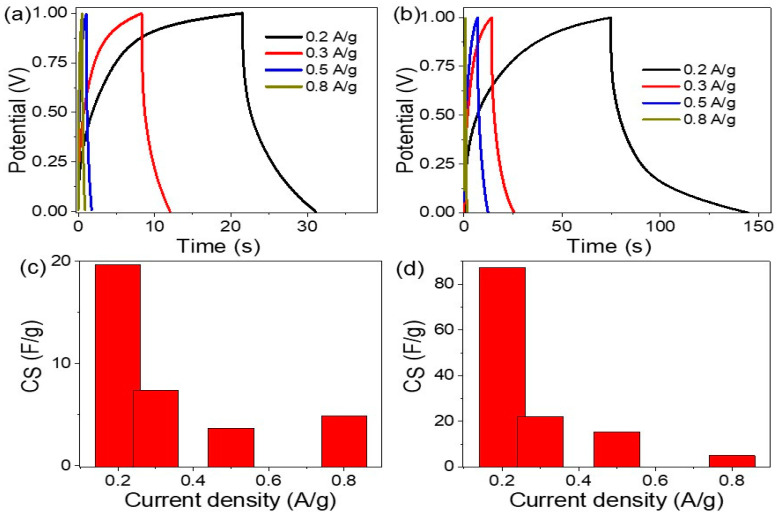
The effect of PB-Ppy nanomaterials on the enhancement of the charge and C_S_ values of R-GO/P2ABT-ML supercapacitor: (**a**,**b**) charge and (**c**,**d**) C_S_ values before and after the PB-Ppy incorporation, correspondingly.

The electrochemical behavior of a R-GO/P2ABT-ML supercapacitor was studied with and without the addition of PB-Ppy nanomaterials using CHI608E measurements and 1.0 M HCl as the electrolyte. The charge/discharge curves were estimated using the proton jump phenomenon, which involves the movement of H^+^ ions through two symmetrical Au plates. The curves represent the charge storage into the plates under different current density (J) values ranging from 0.2 to 0.8 A/g (Figure 3).

The results indicate that as the J value increases, the charge time decreases, indicating limited charge storage at higher J values [37,38,39]. On the other hand, at lower current densities, a longer charge time is observed, which suggests a higher capacitance for the pseudo capacitor. Additionally, the inclusion of PB-Ppy nanomaterials results in an enhancement of the charge time due to its high semi-conductivity values and morphology behavior, which play a role in charge storage.

The specific capacitance (C_S_) values of the supercapacitor were calculated using Equation (2) [40,41], which takes into account the mass (m), current, time (Δt), and potential (ΔV). C_S_ values were calculated for both R-GO/P2ABT-ML and R-GO/P2ABT-ML/PB-Ppy nanocomposites at different J ranging from 0.2 to 0.8 A/g. At a current density of 0.2 A/g, the C_S_ values are 19.6 F/g and 92 F/g for R-GO/P2ABT-ML and R-GO/P2ABT-ML/PB-Ppy, respectively. These results indicate a significant enhancement in the C_S_ values upon the addition of PB-Ppy nanomaterials to the composite:

(2) $$C_{s}=4I.\Delta t/\Delta V.m$$

Equation (3) [40,41] was utilized to calculate the energy density (E values) by taking into account the minimum and maximum potential values of the potential windows. Prior to the addition of PB-Ppy nanomaterials, the E value was computed to be 1.18 W.h.kg^−1^. However, it significantly increased to 5.43 Wh.kg^−1^ after their integration into the supercapacitor:

(3) $$E=0.5C_{s}.\;(V_{max\;}^{2}-V_{min}^{2})/3.6$$

**Figure 4:**
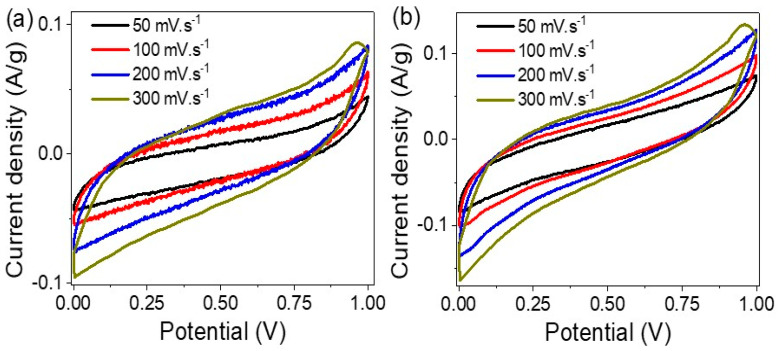
The CV study for the R-GO/P2ABT-ML supercapacitor using 1.0 M HCl in the (**a**) absence and (**b**) presence of PB-Ppy nanomaterials.

The cyclic voltammetry of the R-GO/P2ABT-ML supercapacitor with and without the addition of PB-Ppy nanomaterials is demonstrated in Figure 4a and Figure 4b, respectively. In both cases, an increase in the area under the curve and the produced J values is observed with the scan rate ranging from 50 to 300 mV s^−1^. However, the greatest enhancement is achieved after the incorporation of PB-Ppy materials. The appearance of oxidation and reduction peaks related to the pseudo capacitance behavior is observed, which is related to the change in the oxidation state of S atoms from P2ABT and N atoms from Ppy during the charge and discharge reaction.

**Figure 5:**
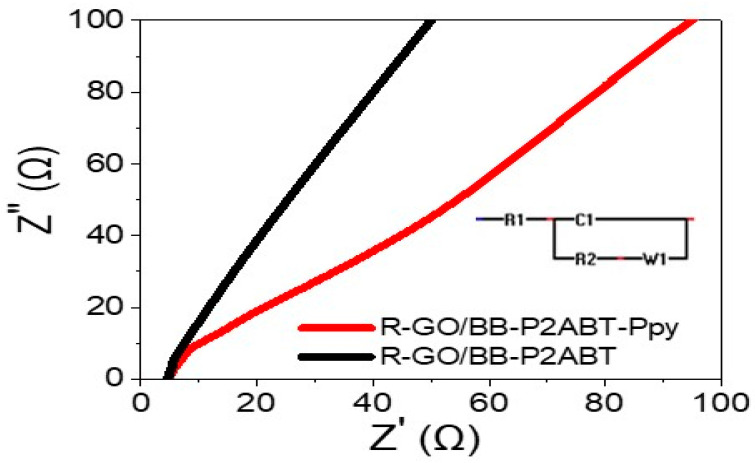
The impedance characteristics of a pseudo capacitor made with R-GO/P2ABT-ML were investigated using 1.0 M HCl in both the absence and presence of PB-Ppy nanomaterials.

The impedance of a supercapacitor made with R-GO/P2ABT-ML and using 1.0 M HCl was studied, both in the absence and presence of PB-Ppy nanomaterials. The results are shown in Figure 5, which illustrates a Nyquist plot. The Randle cell represents the behavior of the supercapacitor, and the R1 (R_S_, series resistance) and R2 (R_CT_) indicate the series and charge transfer resistance [7,42,43], respectively. In the absence of PB-Ppy nanomaterials, the values of R1 and R2 are 4.63 and 0.37 Ω, respectively. However, in the presence of PB-Ppy nanomaterials, the values of R1 and R2 are 4.64 and 0.6 Ω, respectively. This indicates the enhancement of charge storage related to the series resistance value.

The small values of resistance after the addition of PB-Ppy materials indicate that there is a great charge transfer, which is facilitated by the conductivity behavior of the PB-Ppy materials. This behavior is confirmed by the smaller semi-circle observed in the Nyquist plot after the addition of PB-Ppy. Overall, these results suggest that the addition of PB-Ppy nanomaterials improves the performance of the supercapacitor, as it reduces the charge transfer resistance and increases the charge transfer efficiency [42,43].

**Figure 6:**
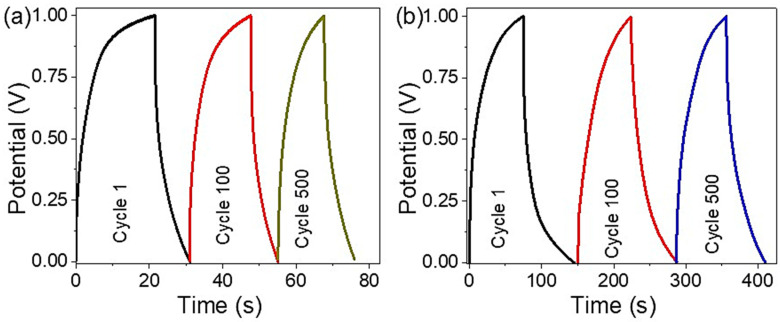
The stability of the R-GO/P2ABT-ML supercapacitor using 1.0 M HCl in the (**a**) absence and (**b**) presence of PB-Ppy nanomaterials.

The stability of R-GO/P2ABT-ML was evaluated in the presence and absence of PB-Ppy nanomaterials, as shown in Figure 6a and Figure 6b respectively. The charging behavior of the supercapacitor was tested for 500 cycles, and the retention coefficient was calculated based on the results presented in Figure 6. The stability of the supercapacitor was found to be 90% and 95.6% in the absence and presence of PB-Ppy nanomaterials, respectively, after 100 cycles. These values decreased to 71% and 85% after 500 cycles, respectively.

The high stability of the supercapacitor confirms the beneficial role of PB-Ppy materials, which can facilitate the charging and discharging process due to their porous structure. This structure allows for easy extraction and shrinkage under repeated charging cycles, thus maintaining the stability of the supercapacitor.

## 4. Conclusions

A highly uniform R-GO/P2ABT-ML nanocomposite is prepared and fully characterized to illustrate the outstanding uniformity in morphological properties. The great layer structure and porosity of this composite, combined with the conductivity behavior, promote its application as a pseudo supercapacitor. Moreover, the insertion of the prepared porous ball structure PB-Ppy is performed to increase the capacitance behavior through a uniform diameter of 150 nm and a highly porous structure.

The pseudo capacitor consists of a uniform nanocomposite paste on both of the two electrodes. At 0.2 A/g, the C_S_ values are 19.6 F/g and 92 F/g for R-GO/P2ABT-ML and R-GO/P2ABT-ML/PB-Ppy, respectively. With the same treatment, the energy density (E) values are 1.18 and 5.43 Wh.kg-1, correspondingly. The stability also has the same enhancement behavior through the incorporation of PB-Ppy, in which the addition of PB-Ppy increases the lifetime of the charge cycles with very noticeable behavior; at 100 and 500 charge cycles, the stability of 95.6 and 85.0%, respectively, which is greater than 89 and 71% without PB-Ppy nanomaterials. The low cost and mass production with the easy fabrication of this pseudo capacitor are instrumental behind the expected synthesis of a prototype of this supercapacitor for commercial applications. 

## Figures and Tables

**Table 1:** The FTIR band position for the characteristic group of PB-Ppy, GO, BB-P2ABT, and R-GO/P2ABT-ML nanocomposite materials.

Materials/Band Position (cm^−1^)				Characteristic Group
PB-Ppy	GO	BB-P2ABT	R-GO/P2ABT-ML	
		3743	3756	N-H
	3400			O-H [28]
		3373	3383	S-H [29]
1702		2570	2601	C-H
	1630			C=O
1622		1514 and 1560	1632	C=C quinoid
1420		1387	1399	C=C benzene
1312		1304	1293	C-N
	1155 and 1049			C-O epoxide [28]
798		757	873	Para disubstituted ring
		578	593	C-H out of plane [30,31,32]

## Data Availability

All data generated or analyzed during this study are included in this article.
